# Unveiling the Role of Pregnancy-Associated Plasma Protein A (PAPP-A) in Pregnancy-Associated Breast Cancer: A Comprehensive Review

**DOI:** 10.7759/cureus.56269

**Published:** 2024-03-16

**Authors:** Archan Patel, Deepika Dewani, Arpita Jaiswal, Lucky Srivani Reddy, Pallavi Yadav, Neha Sethi

**Affiliations:** 1 Obstetrics and Gynecology, Jawaharlal Nehru Medical College, Datta Meghe Institute of Higher Education and Research, Wardha, IND

**Keywords:** therapeutic targeting, diagnostic implications, cancer progression, tissue remodeling, papp-a, pregnancy-associated breast cancer

## Abstract

Pregnancy-associated breast cancer (PABC) presents unique challenges due to its occurrence during or shortly after pregnancy. Pregnancy-associated plasma protein A (PAPP-A) has emerged as a potential biomarker and regulator in PABC. This comprehensive review examines the role of PAPP-A in PABC, highlighting its involvement in tissue remodeling and cancer progression. Molecular mechanisms linking PAPP-A to breast cancer, including signaling pathways and interactions with other molecules, are explored. The review also discusses the diagnostic and therapeutic implications of PAPP-A dysregulation in PABC, emphasizing the need for further research to elucidate underlying mechanisms and develop targeted therapies. Collaborative efforts among researchers, clinicians, and industry stakeholders are essential for translating findings into clinically relevant interventions to improve outcomes for PABC patients.

## Introduction and background

Pregnancy-associated breast cancer (PABC) refers to breast cancer diagnosed during or within a defined period after pregnancy, typically within five years postpartum. PABC poses unique challenges due to the physiological changes that occur during pregnancy and lactation, impacting both diagnosis and treatment strategies [1]. Pregnancy-associated plasma protein A (PAPP-A) is a metalloproteinase enzyme primarily known for modulating insulin-like growth factor (IGF) signaling during pregnancy. The placenta secretes it and regulates fetal growth and development [2].

Investigating the role of PAPP-A in PABC is vital due to emerging evidence suggesting its involvement in breast cancer development and progression. Understanding the interplay between PAPP-A and breast cancer biology could provide valuable insights into the pathogenesis of PABC and potentially lead to the identification of novel diagnostic and therapeutic targets [3]. This comprehensive review aims to explore the current state of knowledge regarding the role of PAPP-A in PABC. By synthesizing existing research findings, we aim to elucidate the mechanisms underlying PAPP-A involvement in PABC and discuss its potential implications for diagnosis, prognosis, and therapeutic interventions.

## Review

Understanding PABC

Definition and Epidemiology

PABC refers to breast cancer diagnosed during pregnancy, lactation, or within the first year postpartum [4-6]. This unique manifestation of breast cancer poses considerable diagnostic and therapeutic challenges due to its occurrence within the context of pregnancy and breastfeeding [4,5]. Epidemiologically, PABC is more prevalent among younger women, primarily due to delayed childbearing and hormonal fluctuations associated with pregnancy and lactation [4]. Studies indicate that PABC patients generally experience a poorer prognosis compared to non-pregnant breast cancer patients, exhibiting higher rates of recurrence and mortality [6,7]. Nonetheless, early detection and appropriate treatment strategies can substantially influence the prognosis of PABC patients [7]. Radiologists play a pivotal role in diagnosing PABC by identifying specific imaging characteristics that differentiate breast cancer from normal physiological changes during pregnancy and lactation [5]. The intricate interaction between hormonal shifts during pregnancy and lactation may contribute to the initiation and progression of breast cancer, underscoring the critical importance of early detection and management approaches for PABC [8].

Risk Factors and Predisposing Factors

**Figure 1:**
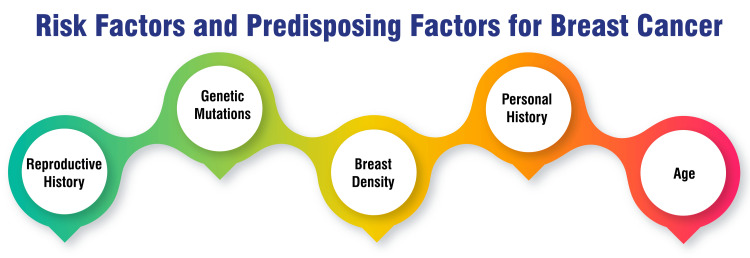
Risk factors and predisposing factors for breast cancer This image has been created by the corresponding author.

Risk and predisposing factors are pivotal in understanding health and disease dynamics. In the realm of pediatric preventive medical care, predisposing factors such as age, gender, and parental education level significantly impact the likelihood of accessing such care. Conversely, enabling factors like having a dedicated healthcare provider enhance the chances of receiving preventive medical attention [9]. Furthermore, protective factors are crucial in mitigating the risk of adverse outcomes and fostering positive health behaviors and results [10]. In scenarios involving anticipatory distress related to painful medical procedures in children, predisposing factors such as genetic predispositions and life events may elevate a child's susceptibility to experiencing heightened distress. Additionally, precipitating factors instigate the onset of distress while perpetuating factors sustain the issue, and present factors operate during the distress-inducing event itself [11]. In infectious diseases, various predisposing factors, including genetic makeup, climatic conditions, age, and overall health status, contribute to an individual's susceptibility to infections. This underscores the necessity of considering a spectrum of biological and environmental factors in devising strategies for disease prevention and control [12]. Comprehending these risk and protective factors is indispensable for formulating effective interventions and approaches to promote health and well-being across diverse populations and circumstances. Risk factors and predisposing factors for breast cancer are shown in Figure 1.

Clinical Presentation and Diagnosis

The clinical presentation of PABC typically manifests with identifiable signs such as a painless palpable mass, skin thickening, or noticeable asymmetrical changes in the breast [13]. Given its frequent occurrence during pregnancy or within the initial year postpartum, PABC often presents as a persistent breast mass, potentially misconstrued as normal physiological changes associated with pregnancy [13]. Additional symptoms commonly observed include skin thickening, nipple discharge (which may be bloody or purulent), lymph node enlargement, and inflammatory alterations upon diagnosis [4]. The aggressive nature of PABC is underscored by factors such as the relatively young age of diagnosis, advanced tumor staging, and a heightened prevalence of hormone receptor negativity [14]. The diagnosis and subsequent management of PABC present considerable challenges, stemming from the imperative to safeguard both maternal and fetal well-being, alongside addressing the psychological ramifications inherent to this distinct condition [15]. Timely assessment and intervention by healthcare providers are essential to circumvent delays linked to poorer prognoses associated with PABC [15].

PAPP-A: structure and function

Molecular Structure of PAPP-A

The molecular architecture of pregnancy-associated plasma protein A (PAPP-A) comprises several essential elements. The PAPP-A subunit features a laminin G-like module situated at the N-terminus, succeeded by a module housing an elongated zinc-binding consensus sequence characteristic of metzincin metalloproteinases like matrix metalloproteinases (MMPs) and ADAMs. Belonging to the pappalysin family alongside PAPP-A2 and ulilysin, PAPP-A's C-terminal region encompasses five short consensus repeat (SCR) modules, also referred to as complement control protein (CCP) modules, alongside a C domain. Notably, the SCR3 and SCR4 modules exhibit binding affinity toward glycosaminoglycans (GAGs), facilitating PAPP-A's interaction with cell surfaces. Moreover, the PAPP-A subunit harbors three Lin12-Notch repeat (LNR) modules, with two located within the proteolytic domain and one within the C domain [16]. Additionally, insights into PAPP-A's structure have been gleaned from its interaction with its endogenous inhibitor, stanniocalcin-2 (STC2). The complex between PAPP-A and STC2 assumes a flexible multidomain conformation characterized by numerous interdomain contacts. Despite inhibition by STC2, the active site cleft within the catalytic domain of the PAPP-A·STC2 complex remains accessible, enabling it to hydrolyze synthetic peptides derived from insulin-like growth factor binding protein-4 (IGFBP-4). This unique mode of proteolytic inhibition holds potential implications for developing tailored pharmaceutical agents aimed at modulating IGF signaling pathways [17]. Moreover, research indicates that PAPP-A serves as a marker of aggressive breast cancer and exerts influence on cancer progression, particularly within the context of pregnancy [18].

Physiological Functions of PAPP-A During Pregnancy

PAPP-A fulfills several vital physiological roles during pregnancy. As a glycoprotein synthesized in the placenta, its concentration in maternal circulation escalates progressively throughout gestation [19]. PAPP-A assumes a crucial function in fetal development by catalyzing the cleavage of IGFBP-4 in the presence of IGF, thereby regulating fetal growth [20]. Furthermore, PAPP-A's significance transcends its role in pregnancy. It has emerged as a notable marker of aggressive breast cancer and has been implicated in cancer progression, particularly in pregnant individuals [18]. Studies indicate that PAPP-A's influence extends beyond pregnancy, with associations noted with adverse pregnancy outcomes and potential therapeutic implications in breast cancer treatment [21,22].

Role of PAPP-A in Tissue Remodeling and Cancer Progression

PAPP-A assumes a pivotal role in tissue remodeling and the progression of cancer, particularly within the realm of breast cancer. Research has underscored PAPP-A as a significant marker of aggressive breast cancer and a driver of cancer advancement, with notable implications, particularly concerning pregnancies [18,23]. The upregulation of PAPP-A has been correlated with aggressive forms of breast cancer and serves as an independent prognostic indicator for early recurrence [23]. Moreover, PAPP-A's association with various facets of tumor development, including initiation, progression, metastasis, and the epithelial-mesenchymal transition (EMT) process, accentuates its critical role in breast cancer pathogenesis [3]. These findings emphasize the imperative of comprehending PAPP-A's involvement in tissue remodeling and its profound impact on cancer progression, particularly within the intricate landscape of breast cancer.

PAPP-A expression in PABC

Overview of PAPP-A Expression in Breast Cancer

PAPP-A is a secreted protease recognized for its significant involvement in breast cancer advancement [18]. Its presence has been consistently observed across nearly all breast cancer specimens, with heightened and extensive-expression notably associated with the luminal B subtype of breast cancer [24]. Overexpression of PAPP-A is prevalent in more than 70% of breast cancer cases and has garnered recognition as a hallmark of aggressive breast cancer [25]. Particularly in PABC tissues, PAPP-A exhibits elevated expression levels and contributes to the progression of breast cancer [26]. Notably, parity has been identified as a predisposing factor, rendering breasts susceptible to the oncogenic effects of PAPP-A, thereby implying that pregnancy-related factors may modulate PAPP-A's role in breast cancer development [25]. The aberrant expression of PAPP-A has been intricately linked to breast cancer progression, rendering it a promising candidate for therapeutic targeting [21]. Serving as an oncogenic protein, PAPP-A assumes a critical function in driving breast cancer advancement, with its relevance particularly underscored in the context of PABC.

Studies Linking PAPP-A Expression With PABC

Numerous studies have established a robust correlation between the expression of PAPP-A and the occurrence of PABC. PAPP-A has been identified as a noteworthy marker of aggressive breast cancer and is implicated in driving cancer progression, particularly within the unique context of pregnancy [18]. The pronounced overexpression of PAPP-A in breast cancer cases, including those associated with PABC, underscores its potential utility as a marker for aggressive disease forms and as a focal point for therapeutic interventions [24,25]. Furthermore, investigations have highlighted the role of parity in heightening breast susceptibility to PAPP-A's oncogenic effects, emphasizing the intricate interplay between pregnancy and breast cancer development [25]. Of particular significance is PAPP-A's involvement in specific breast cancer subtypes, such as triple-negative breast cancer (TNBC), further accentuating its pivotal role in breast cancer pathogenesis [3]. Additionally, aberrant expression of PAPP-A has been observed in breast cancer cell lines, suggesting its active participation in disease progression and its potential suitability as a target for intervention [21]. Collectively, these findings underscore the substantial contribution of PAPP-A to PABC and its promise as both a valuable biomarker and a target for therapeutic intervention in breast cancer management.

Mechanisms Underlying PAPP-A Dysregulation in PABC

The dysregulation of PAPP-A in PABC is characterized by its significant expression in PABC tissues, underscoring its involvement in cancer progression, particularly pertinent in the context of pregnancy [18,26]. Notably, PAPP-A exhibits marked overexpression in more than 70% of breast cancer cases, with a notable focus on PABC instances, thus positioning it as a potential marker for aggressive forms of breast cancer [21]. Furthermore, PAPP-A has been identified as playing a pivotal role in TNBC, a subtype renowned for its aggressiveness and unfavorable prognosis [3]. Experimental investigations have shed light on the intricate regulatory role of PAPP-A in cancer development and progression. Manipulating PAPP-A expression has been demonstrated to impact mitosis, further implicating its involvement in the mechanisms underlying cancer pathogenesis [27].

Molecular mechanisms of PAPP-A in breast cancer development

Signaling Pathways Associated With PAPP-A in Breast Cancer

MMP-dependent pathways: PAPP-A's involvement in breast cancer cell invasion and growth is closely linked to MMP-dependent mechanisms [23]. MMPs are a family of enzymes crucial for degrading ECM proteins, essential for tissue remodeling and cancer progression. In breast cancer, PAPP-A enhances MMP activity, facilitating the breakdown of ECM barriers. This degradation promotes cancer cell invasion into surrounding tissues and facilitates tumor growth and metastasis.

MMP-independent pathways: Besides MMP-dependent mechanisms, PAPP-A can stimulate breast cancer cell invasion and growth through MMP-independent pathways [23]. These pathways involve the activation of various signaling molecules and transcription factors, such as growth factor receptors and nuclear factors, which regulate cell proliferation, survival, and migration. PAPP-A's influence on these pathways enhances breast cancer aggressiveness by promoting cellular processes necessary for tumor progression independently of MMP activity.

Wound healing-related pathways: The signaling pathways implicated in PAPP-A's role in breast cancer progression resemble those involved in wound healing and the creation of tumor-promoting microenvironments [25]. PAPP-A activates growth factors, cytokines, and chemokines crucial for wound healing processes but can also contribute to cancer cell proliferation, migration, and survival. By mimicking wound healing signals, PAPP-A creates a conducive environment for tumor growth and invasion within the breast tissue.

Tumor-promoting microenvironments: PAPP-A's implication in postpartum breast cancer development stems from its ability to influence breast cancer cell behavior within tumor-promoting microenvironments [23]. These microenvironments are characterized by activating various signaling pathways that support cancer progression and metastasis. PAPP-A enhances the aggressiveness of breast cancer cells within these microenvironments, promoting invasion into surrounding tissues and facilitating the establishment of metastatic lesions. Thus, PAPP-A plays a critical role in shaping the tumor microenvironment to favor breast cancer progression following pregnancy.

Interaction of PAPP-A With Other Molecules in Breast Cancer Progression

PAPP-A and IGF1: PAPP-A's interaction with insulin-like growth factor 1 (IGF1) represents a significant axis in breast cancer development. IGF1 is an essential hormone involved in regulating cell growth and differentiation, and its dysregulation has been linked to various cancers, including breast cancer. Research in rodent models suggests that PAPP-A acts as an oncogene, and its interaction with IGF1 provides insights into potential regulatory mechanisms [28]. This interaction likely amplifies the proliferative and survival signals mediated by IGF1, contributing to the oncogenic properties of PAPP-A in breast cancer. Moreover, the interplay between PAPP-A and IGF1 may influence other pathways crucial for tumor progression, such as PI3K/AKT and MAPK/ERK signaling, further enhancing breast cancer cell proliferation, survival, and metastasis.

PAPP-A and MMPs: PAPP-A's involvement in breast cancer cell invasion and growth is closely linked to MMPs, enzymes responsible for degrading the extracellular matrix (ECM) components. MMPs facilitate cancer cell invasion by breaking down ECM barriers, enabling cells to migrate into surrounding tissues and promoting metastasis. The interaction between PAPP-A and MMPs likely contributes to breast cancer cells' invasive and metastatic properties [25]. PAPP-A may enhance MMP activity directly or indirectly by activating downstream signaling pathways involved in MMP regulation. This interaction potentiates the remodeling of the ECM, creating a favorable microenvironment for tumor progression. Additionally, MMP-independent mechanisms mediated by PAPP-A may also influence breast cancer cell behavior, suggesting a multifaceted role for PAPP-A in promoting tumor aggressiveness. Understanding the interplay between PAPP-A and MMPs could unveil novel therapeutic targets for inhibiting breast cancer invasion and metastasis.

Implications of PAPP-A Dysregulation in PABC Prognosis and Treatment

The dysregulation of PAPP-A carries significant implications for both the prognosis and treatment of PABC. PAPP-A serves as a marker of aggressive breast cancer and actively promotes cancer progression, particularly within the context of PABC [18]. Its role in breast cancer is pivotal, notably in the case of TNBC [3]. Anomalies in PAPP-A expression have emerged as significant factors in breast cancer advancement, indicating its potential as a promising therapeutic target [21]. The prognosis of PABC remains inadequately defined, necessitating further research to comprehensively understand the clinical characteristics, pregnancy outcomes, and ovarian function among PABC patients [29]. The consistent detection of PAPP-A in nearly all breast cancer specimens, coupled with its heightened and more extensive-expression correlating with luminal B breast cancer, underscores the pivotal role of PAPP-A in breast cancer progression. Moreover, it suggests that PAPP-A could serve as a valuable diagnostic and prognostic marker for PABC [24].

Clinical implications and therapeutic targeting

Diagnostic and Prognostic Potential of PAPP-A in PABC

PAPP-A demonstrates promising diagnostic and prognostic value in PABC. Numerous studies have identified PAPP-A as a marker indicative of aggressive breast cancer and have elucidated its role in driving disease progression, particularly within pregnancy-related contexts [18]. Notably, the expression of PAPP-A in breast cancer specimens, notably in luminal B subtypes, correlates with heightened disease severity and extent of progression [24]. This underscores the significance of PAPP-A in the trajectory of breast cancer and highlights its potential as a target for therapeutic interventions [21]. In the management of PABC, treatment strategies are tailored to preserve the pregnancy while carefully weighing the risks posed to the developing fetus [30]. Therefore, comprehending the diagnostic and prognostic implications of PAPP-A in PABC is paramount for enhancing patient outcomes and devising targeted therapeutic approaches.

Therapeutic Strategies Targeting PAPP-A in PABC

Several therapeutic strategies have been proposed to target PAPP-A in PABC, leveraging its role in breast cancer progression and its potential as a therapeutic target [21]. One avenue involves the development of small molecule inhibitors tailored to target PAPP-A specifically. These inhibitors hold promise in blocking the oncogenic effects instigated by PAPP-A, thereby impeding or even arresting breast cancer progression [31]. Another therapeutic approach entails identifying and targeting the downstream signaling pathways activated by PAPP-A within breast cancer cells. By inhibiting these pathways, it may be feasible to disrupt the cancer-promoting effects exerted by PAPP-A [26]. Furthermore, recent research indicates that PAPP-A-driven PABC might respond favorably to therapies aimed at targeting DDR2, a protein implicated in the oncogenic mechanisms orchestrated by PAPP-A. This discovery opens avenues for developing novel therapeutic strategies specifically tailored to target DDR2 in PAPP-A-driven PABC [25].

Challenges and Future Directions in PAPP-A-Based Therapy for PABC

Identifying novel therapeutic targets: While PAPP-A holds promise as a therapeutic target in PABC, further exploration is warranted to uncover additional proteins or pathways involved in PAPP-A-driven PABC. Recent studies have hinted at DDR2, a protein implicated in cell migration and invasion, as a potential novel therapeutic target in PAPP-A-driven PABC [25]. Investigating such targets could broaden our understanding of the complex molecular landscape of PABC and unveil new avenues for targeted interventions aimed at halting disease progression.

Developing targeted therapies: The development of targeted therapies tailored to address PAPP-A or its downstream effects is paramount for enhancing the treatment outcomes of PABC patients. This endeavor may entail the design of small molecules or antibodies capable of inhibiting PAPP-A's activity or interfering with its interactions with other proteins [32]. By directly targeting PAPP-A or its downstream mediators, these therapies have the potential to mitigate the oncogenic effects driven by PAPP-A and impede breast cancer progression.

Overcoming resistance to therapy: Resistance to therapy poses a significant challenge in cancer treatment, including PABC management. Understanding the underlying resistance mechanisms to PAPP-A-based therapy and devising strategies to overcome it is imperative for enhancing treatment efficacy [33]. Efforts to decipher the intricacies of resistance mechanisms could lead to the development of innovative approaches to circumvent or counteract resistance, thereby prolonging the effectiveness of PABC treatment modalities.

Personalized medicine: Given the variability in PAPP-A expression across different breast cancer subtypes, personalized medicine approaches tailored to patients' PAPP-A expression and other molecular markers may offer superior efficacy in treating PABC [34]. By incorporating patient-specific characteristics, such as PAPP-A expression levels, into treatment decision-making, personalized medicine strategies have the potential to optimize treatment outcomes and minimize adverse effects in PABC patients.

Combination therapies: Combining PAPP-A-based therapies with other targeted therapies or immunotherapies holds promise for augmenting the overall effectiveness of treatment and mitigating the risk of resistance [35]. Combining therapies can enhance tumor control and improve patient outcomes in PABC by leveraging synergistic interactions between different treatment modalities. Exploring rational combinations of PAPP-A-targeted therapies with other therapeutic agents represents a promising strategy to maximize treatment efficacy and overcome therapeutic resistance in PABC.

## Conclusions

In conclusion, the review highlights the pivotal role of PAPP-A in PABC. PABC poses distinct challenges due to its occurrence during or shortly after pregnancy, necessitating a comprehensive understanding of its underlying mechanisms. Through this review, it becomes evident that PAPP-A is a potential biomarker and a key regulator in PABC development. Its involvement in tissue remodeling and cancer progression underscores its significance. Future research should delve deeper into elucidating the precise mechanisms driving PAPP-A dysregulation in PABC, considering genetic, epigenetic, and environmental factors. Moreover, exploring the interplay between PAPP-A and hormonal fluctuations during pregnancy and postpartum could provide valuable insights into breast cancer development. Advancements in diagnostic methods to accurately measure PAPP-A levels and targeted therapies aimed at modulating its activity hold promise for improved early detection and personalized treatment strategies. Longitudinal studies are warranted to ascertain the prognostic value of PAPP-A and its utility as a predictive marker for treatment response. Collaboration among researchers, clinicians, and industry stakeholders is crucial for translating these findings into clinically relevant interventions that can enhance outcomes for PABC patients.
